# Infected pancreatic necrosis complicating severe acute pancreatitis in critically ill patients: predicting catheter drainage failure and need for necrosectomy

**DOI:** 10.1186/s13613-022-01039-z

**Published:** 2022-08-02

**Authors:** Charlotte Garret, Marion Douillard, Arthur David, Morgane Péré, Lucille Quenehervé, Ludivine Legros, Isabelle Archambeaud, Frédéric Douane, Marc Lerhun, Nicolas Regenet, Jerome Gournay, Emmanuel Coron, Eric Frampas, Jean Reignier

**Affiliations:** 1 Service de Médecine Intensive Réanimation, Centre Hospitalier Universitaire de Nantes, 44000 Nantes, France; 2 Institut des Maladies de L’Appareil Digestif, Centre Hospitalier Universitaire de Nantes, 44000 Nantes, France; 3 Radiologie, Centre Hospitalier Universitaire de Nantes, Nantes, France; 4 Plateforme de Méthodologie et Biostatistique, Direction de la Recherche, Centre Hospitalier Universitaire de Nantes, 44000 Nantes, France; 5 Service d’Hépatogastroentérologie, Centre Hospitalier Universitaire de Brest, 29200 Brest, France; 6 Service d’Hépatogastroentérologie, Centre Hospitalier Universitaire de Rennes, 35203 Rennes, France

**Keywords:** Acute pancreatitis, Infected necrosis, Necrosectomy, Intensive care, Organ failure, Catheter drainage

## Abstract

**Background:**

Recent guidelines advocate a step-up approach for managing suspected infected pancreatic necrosis (IPN) during acute pancreatitis. Nearly half the patients require secondary necrosectomy after catheter drainage. Our primary objective was to assess the external validity of a previously reported nomogram for catheter drainage, based on four predictors of failure. Our secondary objectives were to identify other potential predictors of catheter-drainage failure. We retrospectively studied consecutive patients admitted to the intensive care units (ICUs) of three university hospitals in France between 2012 and 2016, for severe acute pancreatitis with suspected IPN requiring catheter drainage. We assessed drainage success and failure rates in 72 patients, with success defined as survival without subsequent necrosectomy and failure as death and/or subsequent necrosectomy required by inadequate improvement. We plotted the receiver operating characteristics (ROC) curve for the nomogram and computed the area under the curve (AUROC).

**Results:**

Catheter drainage alone was successful in 32 (44.4%) patients. The nomogram predicted catheter-drainage failure with an AUROC of 0.71. By multivariate analysis, catheter-drainage failure was independently associated with a higher body mass index [odds ratio (OR), 1.12; 95% confidence interval (95% CI), 1.00–1.24; *P* = 0.048], heterogeneous collection (OR, 16.7; 95% CI, 1.83–152.46; *P* = 0.01), and respiratory failure onset within 24 h before catheter drainage (OR, 18.34; 95% CI, 2.18–154.3; *P* = 0.007).

**Conclusion:**

Over half the patients required necrosectomy after failed catheter drainage. Newly identified predictors of catheter-drainage failure were heterogeneous collection and respiratory failure. Adding these predictors to the nomogram might help to identify patients at high risk of catheter-drainage failure.

ClinicalTrials.gov number: NCT03234166.

**Supplementary Information:**

The online version contains supplementary material available at 10.1186/s13613-022-01039-z.

## Background

Optimal treatment of infected pancreatic necrosis (IPN) is crucial in critically ill patients with acute pancreatitis. The minimally invasive step-up approach consists in percutaneous or endoscopic drainage followed, if necessary, by minimally invasive retroperitoneal necrosectomy or endoscopic necrosectomy. Compared to open necrosectomy, this approach has been shown to effectively remove necrotic foci and to improve patient outcomes [1–3]. However, mortality in patients with severe acute pancreatitis remains high, at 16–30% [1, 3, 4]. Ineffective drainage is a leading cause of poor outcomes, despite the introduction of percutaneous and endoscopic techniques [5, 6]. However, the first drainage intervention fails to ensure adequate necrotic-tissue removal in nearly half the patients, who must then undergo necrosectomy [1, 3, 7–9]. Identifying patients who will need necrosectomy after initial drainage is challenging, as reliable predictors and clear recommendations are lacking [10–12]. During the course of AP, prolonged antimicrobial therapy is associated with an increased risk of antibiotic-resistant bacteria selection [13, 14] and fungal infection [15]. To date, there is no clear recommendation about whether antibiotics should be given immediately or postponed in patients with IPN [16–18]. A study showed that male sex, multiorgan failure, a higher percentage of necrotic pancreatic tissue, and collection heterogeneity predicted catheter-drainage failure (CDF) [8]. These four factors were used to develop a nomogram for predicting successful initial catheter drainage. Success rates were 91% with the best scores and 2% with the worst scores. However, this nomogram has been assessed only in Dutch centers with considerable expertise in managing acute pancreatitis, and external validation is thus lacking.

The primary objective of this multicenter retrospective observational study was to assess the Dutch nomogram for predicting CDF in patients with suspected IPN. The secondary objective was to identify other potential predictors of CDF.

## Materials and methods

The appropriate ethics committee (*Groupe Nantais d'Éthique dans le Domaine de la Santé*) approved the study (#2017–10-05). In accordance with French legislation on research using anonymized retrospective data, informed consent was not required.

### Study design

Adults (≥ 18 years of age) admitted between January 2012 and December 2016 to any of three French university hospitals (in Angers, Nantes, and Rennes) were identified by searching the hospital databases for codes K85.0 to K85.9 in the International Classification of Diseases-10th revision then selecting patients who required ICU admission for acute pancreatitis.

We screened consecutive patients who underwent primary catheter drainage for suspected IPN, either percutaneously or endoscopically. We included patients with definite IPN defined as computed tomography (CT) evidence of a collection containing extraluminal gas and/or a positive culture of pancreatic tissue obtained by fine-needle aspiration, drainage, or necrosectomy. Patients with catheter drainage procedures performed after necrosectomy or abdominal surgery were not included. All CTs performed before the first drainage procedure were reviewed by a blinded expert radiologist (AD) to assess items included in the nomogram (percentage of pancreatic necrosis and heterogeneous collection).

### Data collection

We recorded demographic data, the Sequential Organ Failure Assessment (SOFA) score at 24 h, organ failure onset during the 48 h after ICU admission and the 48 h preceding the first drainage procedure (a SOFA subscore ≥ 2 in any component defines failure of the relevant organ; Additional file 1: Digital Content S1, [19]), and the initial CT findings including the CT Severity Index. We also recorded the following data on management: timing and type of procedures (percutaneous or endoscopic transluminal drainage, endoscopic or surgical necrosectomy); type and duration of antimicrobial therapy before and after drainage; and findings from microbiological studies of blood and IPN specimens, including the presence of multidrug-resistant (MDR) bacteria. Vital status and hospital and ICU stay lengths were collected. Recorded findings by CT performed before the first drainage procedure included location and percentage of necrosis, degree of collection encapsulation, collection contents, presence of gas, and portosplenomesenteric venous thrombosis or narrowing (Additional file 1: Digital Content S2) [8, 20].

### Management

When IPN was suspected, percutaneous or endoscopic drainage was performed. The choice between these two methods was made by the attending intensivist based on collection location by CT, operator preference, and radiology or endoscopy suite availability. Empirical broad-spectrum antibiotic therapy was started before microbiological specimen collection in patients with septic shock or persistent organ failure. When cultures recovered one or more organisms, the antibiotics were adjusted according to susceptibility test results, with narrowing of the spectrum to the extent possible. Patients with no improvement after the first drainage procedure underwent CT (typically after 72 h) followed, if necessary by a second drainage procedure and/or by endoscopic or surgical necrosectomy. The choice of antibiotics and their duration of administration was at the discretion of the attending intensivist.

### Definitions of drainage success and failure

Percutaneous drainage was defined as percutaneous catheter placement into the retroperitoneum and/or pelvis and endoscopic drainage as the introduction of two double-pigtail stents or self-expandable metal stents between the stomach or duodenum and the acute collection or wall of necrosis [2]. Endoscopic necrosectomy was defined as placement of self-expandable, metal stents ≥ 15 mm diameter providing endoscopic access for direct necrosectomy using debridement techniques [2].

Catheter drainage success was defined as survival without necrosectomy, regardless of the number of drainage procedures [8]. CDF was defined as death, and/or necrosectomy after drainage due to lack of improvement.

### Statistics

Baseline characteristics of the overall population were expressed as frequencies (percentages) for categorical variables, mean ± standard deviation (SD) for normally distributed continuous data, and median (25th–75th percentile) for skewed continuous data. To compare baseline values between the groups with successful and failed drainage, we applied Student’s test for continuous variables and the *χ*^2^ test for categorical variables. We performed multivariate logistic regression analysis to identify factors associated with IPN. All variables entered into the model were recorded before the need for necrosectomy was identified. The following variables were assessed: age, sex, body mass index (BMI), pancreatic necrosis > 50%, heterogeneous collection, and respiratory failure onset within 24 h before the first drainage procedure. Variables yielding *P* values ≤ 0.2 by univariate analysis were tested, and forward variable selection was performed. The odds ratios (ORs) with their 95% confidence intervals (95% CIs) were computed. All tests were two-tailed. *P* values lower than 0.05 were considered significant.

We plotted the receiver operating characteristics curve of drainage failure predictors used in the Dutch nomogram [8] (Additional file 1: Digital Content S3) and computed the area under the curve (AUROC).

The data were analyzed using SAS software (version 9.4, Cary, NC).

## Results

### Patients and clinical outcomes

**Fig. 1:**
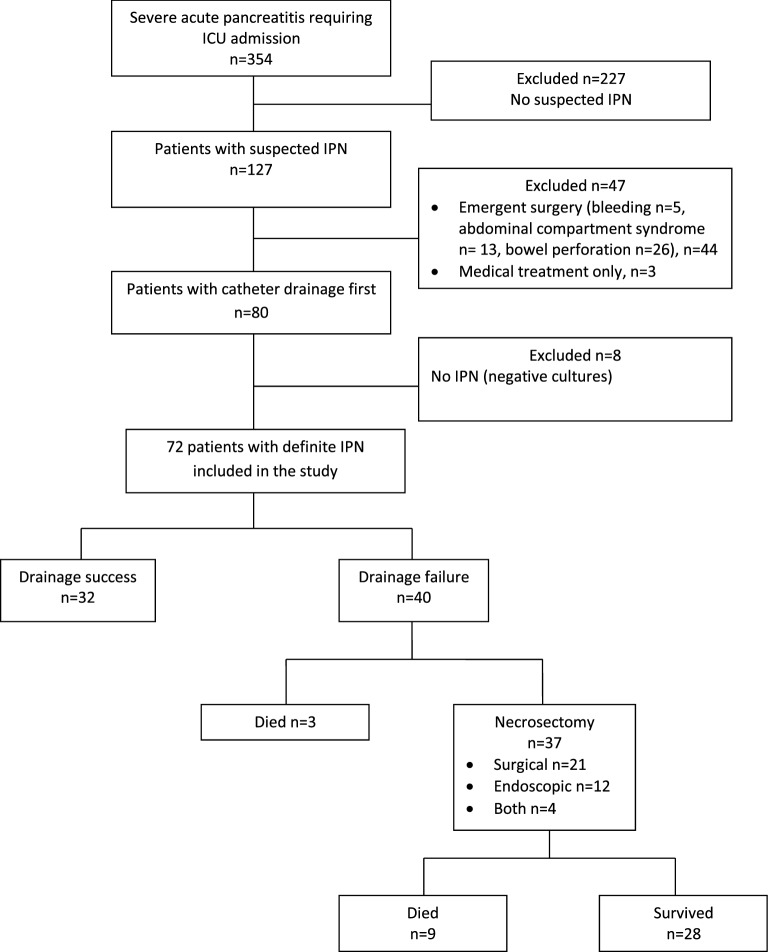
Patient flowchart. *IPN* infected pancreatic necrosis

Figure 1 is the patient flowchart. Table 1 reports the main characteristics and outcomes of the study patients.

**Table 1:** Baseline characteristics and outcomes of the study patients

	Overall population *n* = 72	Drainage success *n* = 32	Drainage failure *n* = 40	*P* value
Baseline characteristics and CTSI				
Age, y, mean (SD)	57 (149)	56 (16)	58 (14)	0.54
Males, *n* (%)	58 (80.6)	26 (81.3)	32 (80)	0.89
BMI, mean (SD)	27.94 (6.5)	25.83 (4.6)	29.65 (7.4)	**0.02**
Cause of pancreatitis, *n* (%)				
Biliary	23 (31.9)	10 (31.2)	13 (32.5)	0.98
Alcohol abuse	25 (34.7)	12 (37.5)	13 (32.5)	
Other^a^	24 (33.3)	10 (31.2)	14 (35)	
SOFA score^b^, median [IQR]	3 [0–12]	2 [0–11]	3 [0–12]	0.39
CTSI, mean (SD)	7 (2)	6 (2)	7 (2)	0.07
Persistent organ failure^c^ after the first 48 h, *n* (%)				
Respiratory failure	17 (23.9)	5 (15.6)	12 (30.8)	0.14
Hemodynamic failure	11 (15.5)	3 (9.4)	8 (20.5)	0.21
Renal failure	8 (11.1)	3 (9.4)	5 (12.5)	0.67
Organ failure^c^ within 24 h before catheter drainage, *n* (%)				
Respiratory failure	29 (40.8)	7 (21.9)	22 (56.4)	**0.004**
Hemodynamic failure	15 (21.1)	5 (15.6)	10 (25.6)	0.3
Renal failure	9 (12.7)	3 (9.4)	6 (15.4)	0.45
Drainage modalities				
Time from diagnosis to drainage (days), median [IQR]	21 [12–29]	18 [9–25]	24 [15–28]	0.06
Percutaneous catheter drainage *n* (%)	52 (72.2)	26 (81.2)	26 (65)	0.13
Endoscopic catheter drainage *n* (%)	20 (27.8)	6 (18.7)	14 (35.)	
Antibiotic therapy before drainage *n* (%)	41 (60.3)	20 (64.5)	21 (56.7)	0.51
Time from antibiotic initiation to drainage (days), mean (SD)	7 (7)	9 (8)	5(5)	0.08
Second drainage (percutaneous or endoscopic)	41 (74.5)	19 (59.4)	22 (95.6)	**0.01**
Overall antibiotic duration during hospital stay (days), median [IQR]	47 [28–77]	38 [26–61]	50 [28–88]	0.17
Microbiological results, *n* (%)				
Bacteremia	39 (54.2)	18 (56.2)	21 (52.5)	0.75
Fungal microorganism	13 (18.1)	5 (15.6)	8 (20.)	0.63
Multidrug- resistant bacterial microorganism	24 (33.3)	7 (21.9)	17 (42.5)	0.06
Complications of acute pancreatitis during ICU stay, *n* (%)				
Bleeding	14 (19.4)	2 (6.2)	12 (30)	**0.011**
Perforation of hollow organ	8 (11.1)	0 (0)	8 (20)	**0.007**
Bowel ischemia	5 (6.9)	0 (0)	5 (12.50%)	0.06
Outcome				
Hospital stay length (days), median [IQR]	70 [43–98]	53 [36–70]	88 [61–114]	**0.001**
Hospital mortality, *n* (%)	12 (16.7)	0 (0)	12 (30)	**0.0007**

*CTSI* computed tomography severity index, *BMI* body mass index; *SOFA* Sequential Organ Failure Assessment. Bold numbers indicate p<=0.05

^a^Hypertriglyceridemia, drugs, endoscopic retrograde cholangiopancreatography, trauma, unidentified

^b^The scale ranges from 0 to 24, with higher scores indicating greater severity of organ dysfunction

^c^Organ failure was defined as a SOFA subscore ≥ 2 for the respiratory, renal, and/or hemodynamic systems

The median time from diagnosis to drainage was 21 [12–29] days and the median number of catheter drainage procedures per patient was 2 [1–3], with no significant between-group difference. Catheter drainage alone was successful in 32 (44%) patients. Necrosectomy after catheter drainage was required in 37 patients, at a median of 44 [36–60] days. Necrosectomy was surgical in 23 (62%) patients and endoscopic in 14 (38%) patients. No patients were treated for abdominal decompression. The remaining 3 patients died before undergoing necrosectomy.

Of the 72 patients, 12 (16.6%) died (Fig. 1). All deaths were related to severe AP. The cause of death was refractory shock with multiorgan failure in 5 (41.6%) patients, bowel ischemia diagnosed by CT or open laparotomy in 4 (30.7%) patients, and refractory abdominal bleeding in 3 (24.9%) patients.

### Dutch nomogram

**Fig. 2:**
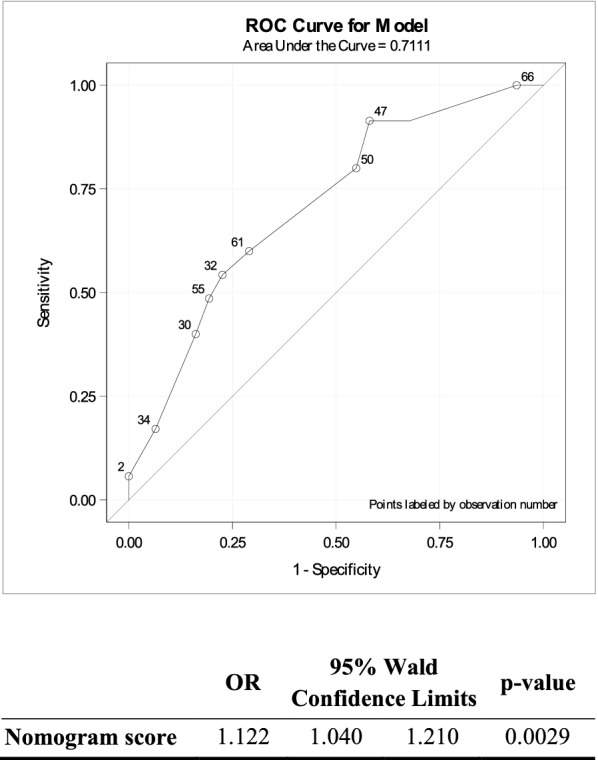
Receiver operating characteristic (ROC) curve of the multivariate regression model for predicting success of catheter drainage in patients with infected pancreatic necrosis using the Dutch nomogram based on male sex, multiorgan failure, percentage of pancreatic necrosis, and density of the collection [8]. The area under the curve was 0.71 (95% confidence interval, 0.5869; 0.8352)

Applying the Dutch nomogram to our cohort after the first drainage procedure resulted in an AUROC of 0.71 (95% CI, 0.58–0.83) (Fig. 2). Higher scores were associated with CDF (OR, 1.12; 95% CI, 1.04–1.21; *P* = 0.002).

### Univariate and multivariate analysis of potential predictors of catheter-drainage failure (CDF)

Table 2 reports the findings from the univariate logistic regression analysis. By multivariate analysis, predictors of CDF were higher BMI (OR, 1.14; 95% CI, 1.01–0.29; *P* = 0.02), heterogeneous collection (OR, 18.84; 95% CI, 2.02–175.86; *P* = 0.01), and respiratory failure 24 h before catheter drainage (OR, 16.76; 95% CI, 1.94–144.4; *P* = 0.01) (Table 3).

**Table 2:** Univariate regression analysis to identify predictors of catheter-drainage failure

	Odds ratio	95% CI	*P* value
Baseline characteristics and CTSI			
Age	1.01	[0.98; 1.04]	0.5388
Male	1.08	[0.33; 3.52]	0.8944
BMI	1.11	[1.01; 1.22]	**0.0246**
SOFA	1.02	[0.94; 1.14]	0.39
Persistent organ failure^a^, after the first 48 h			
Respiratory failure	2.40	[0.74; 7.75]	0.1431
Hemodynamic failure	2.49	[0.60; 10.32]	0.2071
Renal failure	1.38	[0.30; 6.27]	0.6760
Complications during the 24 h before first drainage			
Respiratory failure	4.62	[1.62; 13.21]	**0.0043**
Hemodynamic failure	1.86	[0.56; 6.15]	0.3078
Renal failure	1.76	[0.40; 7.67]	0.4531
Data on last CT before drainage			
CTSI before drainage	1.20	[0.98; 1.48]	0.07
% of pancreatic necrosis			
Pancreatic necrosis < 30%	0.46	[0.17; 1.25]	0.12
Pancreatic necrosis ≥ 30 < 50%	0.69	[0.19; 2.55]	0.58
Pancreatic necrosis ≥ 50%	3.47	[1.07; 11.19]	**0.03**
Heterogeneous collection	2.44	[0.84; 7.06]	0.10
Gas bubbles in collection on CT	1.23	[0.38; 4.06]	0.72
Acute necrotic collection	0.00	[0.00; I]*	0.9799
Walled-off necrosis	1.87	[0.32; 11.00]	0.4883
PSM vein thrombosis	0.98	[0.34; 2.84]	0.96
PSM vein narrowing	2.33	[0.86; 6.29]	0.09
Drainage modalities			
Time from diagnosis to drainage (per additional day)	1.04	[1.00; 1.07]	0.0663
Antibiotic therapy ≥ 48 h before drainage	0.72	[0.27; 1.93]	0.5154
Second drainage (percutaneous or endoscopic)	**15.05**	**[1.80; 125.95]**	**0.0124**

*95% CI* 95% confidence interval, *CTSI* computed tomography severity index, *BMI*: body mass index, *SOFA* Sequential Organ Failure Assessment, *PSM*: portosplenomesenteric. Bold numbers indicate p<=0.05

^a^Organ failure was defined as a SOFA subscore ≥ 2 for the respiratory, renal, and/or hemodynamic components

**Table 3:** Multivariate analysis to identify predictors of catheter drainage failure

	aOR [95% CI]	*P* value
Age (per additional year)	1.00 [0.96; 1.05]	0.89
Male sex	3.24 [0.51; 20.73]	0.21
Body mass index (per additional 1 kg·m^−2^)	1.14 [1.01; 1.29]	**0.02**
Heterogeneous collection	18.84 [2.02; 175.86]	**0.01**
Respiratory failure onset within 24 h before first drainage	16.76 [1.94; 144.40]	**0.01**

*aOR* adjusted odds ratio, *95% CI* 95% confidence interval. Bold numbers indicate p<=0.05

Additional file 1: Digital Content S4 details the microbiological findings. Of the 72 patients, 51 (71%) had more than one organism recovered, including at least one Gram-negative organism. At least one blood culture was positive at some point in 39 (54.1%) patients. *Escherichia coli* and *Enterococcus* were the most commonly recovered organisms. *Candida albicans* or *Candida glabrata* was identified in 13 (18%) patients. Infection with MDR microorganisms was found in 24 (33.3%) patients. MDR bacteria were more common in the group with CDF, although the difference was not statistically significant (45.5% vs. 21.8%; *P* = 0.06) (Table 1). The antibiotics used are detailed in the Additional file 1: Digital Content S5.

## Discussion

This is the first study investigating the external validity of the Dutch nomogram designed to predict the efficacy of catheter drainage as the first drainage procedure in patients with IPN. The nomogram produced an AUROC of 0.71, similar to the 0.76 value reported by its designers [8]. Other CDF predictors identified by our study were higher BMI, heterogeneous collection on the last CT performed before catheter drainage, and respiratory failure onset within 24 h before catheter drainage.

Our 44% proportion of patients with successful catheter drainage is consistent with the literature [1, 6, 8, 21], as is the approximately 21-day interval from acute pancreatitis diagnosis to first catheter drainage [22, 23]. The 44-day time from admission to first necrosectomy can be explained by the prior performance of a second drainage procedure in three-quarters of patients. In addition, time from admission to first necrosectomy was similar to that in previous studies of the step-up approach [3, 24].

Interestingly, endoscopic drainage was performed in a higher proportion of patients (28%) than in the seminal Dutch study [8]*.* One possible explanation is the later recruitment in our study, at a time when interventional endoscopy was more widely available. Also, our cohort more closely reflects real-life conditions.

The nomogram predicted CDF similarly as in the seminal study [8], which was conducted in patients prospectively included in two randomized controlled trials. The recruitment period for one of these trials (PROPATRIA) [25] was 2004–2007, before the step-up approach was widely used, at a time when surgical necrosectomy was still the reference standard treatment for IPN. Another Dutch study [1] compared the minimally invasive step-up approach to open necrosectomy. Thus, our retrospective multicenter cohort study may better reflect real-life practice, thereby supporting the potential usefulness of the Dutch nomogram. However, we identified additional risk factors that might improve decision-making at the bedside.

Respiratory failure onset within 24 h before catheter drainage was strongly associated with CDF by multivariate analysis. To our knowledge, either single-organ failure or multiorgan failure has been assessed in previous studies, without specifying the type of organ failure, i.e., hemodynamic, renal, or respiratory. Multiorgan failure before the first catheter drainage predicted CDF in several previous studies [8, 9, 22, 26, 27]. Respiratory failure correlates with the severity of acute pancreatitis and reflects acute respiratory distress syndrome due to severe systemic inflammation [28]. IPN is uncommon during the first 2 weeks of acute pancreatitis [4, 10, 11, 20]. The type and number of organ failures may better reflect severity when recorded within 24 h of the first drainage procedure rather than at ICU admission. In our study, respiratory failure within 24 h before the first drainage procedure strongly predicted CDF failure. Thus, recent respiratory failure onset may be a good indicator that necrosectomy will be required promptly. Further studies of the type and timing of organ failures, notably respiratory failure, would be of interest. Another new finding from our study is the significant association of higher BMI with CDF. Obesity correlated with disease severity, local complications such as walled-off necrosis, and mortality in several studies, but the potential link with catheter drainage outcomes was not assessed [29, 30]. Obesity raises technical challenges with drain placement and maintenance, especially when drainage is percutaneous. That percutaneous catheter drainage was performed in most patients (72%) may explain the association between obesity and CDF.

In contrast to the study describing the nomogram [8], neither male sex nor percentage of necrosis predicted CDF. However, a possible explanation is the 80.5% proportion of males in our population compared to only 66% in the previous study. In three other studies, male sex also failed to predict CDF [21, 22, 27]. The percentage of necrosis predicted CDF in two previous studies [8, 27]. This variable was not predictive by multivariate analysis in our study or two earlier studies [21, 22]. Similarly, a heterogenous collection was strongly associated with CDF by multivariate analysis in the seminal study [8] and two other studies [21, 22]. The presence on CT images of several different attenuations usually indicates solid necrotic debris within the collection, which can be difficult to eliminate by a single catheter drainage procedure. In IPN, the persistence of infected debris within the collection is strongly associated with a higher risk of sepsis and suggests a need for prompt necrosectomy. Quantitative assessments of CT attenuation indicated that values above 30 HU, reflecting solid components, were associated with CDF [21, 31, 32]. Conversely, attenuation below < 20 HU, reflecting a liquid component, was associated with a high probability of successful catheter drainage. However, these measurements require CT without contrast agent. In our patients, most of the CT scans done before the first catheter drainage used a contrast agent, making us unable to assess attenuation. Given the potential promise of collection attenuation as a predictor of catheter drainage outcomes, future studies of this parameter would be of interest.

The main limitation of our study is the retrospective observational design, which is inherently associated with bias and cannot demonstrate causality. Nonetheless, the sample size obtained via the multicenter recruitment was sufficient to allow a preliminary evaluation of the Dutch nomogram under real-life conditions. We had no information on the type or route of nutritional support or on drain size. We had no information on the type or route of nutritional support or on drain size. Some predictors may have gone unidentified due to missing data, such as intra-abdominal hypertension. However, several factors can influence intra-vesical pressure measurement [33] Another limitation is the lack of a standardized therapeutic algorithm, which may have induced bias via several mechanisms, such as the choice of one method over another. Also, the procedures and types of devices were at the discretion of the physicians, and operating-room availability may have led to one type of intervention being performed rather than another. Furthermore, we tested nomogram performance with the step-up approach. In our study, there was a trend toward a longer time from diagnosis to drainage in the drainage-failure group, whereas postponed drainage was recently reported to be associated with fewer interventions [18]. Currently, there is no clear consensus on the optimal timing of drainage [2, 17, 18, 34, 35] and further studies are needed to clarify this point. Finally, we excluded 44 (34.6%) patients who required emergency laparotomy before drainage, a proportion similar to that in two randomized controlled trials of the step-up approach (28% [1] and 42% [3]). This point may explain the relatively low overall mortality. Nonetheless, the mortality rate in our cohort is consistent with those in the two above-mentioned trials [1, 3].

## Conclusion

Our findings support the usefulness of the Dutch nomogram for predicting CDF in ICU patients with IPN. Adjustments to this nomogram might improve accuracy, and validation in a larger prospective cohort is needed. Our results and those of earlier studies indicate that additional predictors of CDF may include organ failure before drainage (notably respiratory failure) and heterogeneous CT collection [8, 10, 21, 22, 27, 32]. These predictors might help to identify patients at high risk for CDF.

## Supplementary Information

**Additional file 1: Digital Content S1.** SOFA score (Vincent et al. 1998 [19]). **Digital Content S2.** Computed tomography (CT) findings according to the revised Atlanta classification [20] and study by Hollemans et al. [8]. **Digital Content S3.** Dutch nomogram [8]. **Digital Content S4.** Microorganism recovered from cultures of specimens of infected pancreatic necrosis (IPN); distribution of multidrug-resistant (MDR) microorganisms. **Digital Content S5.** Antibiotics used before drainage (*n* = 41) then during the course of infected pancreatic necrosis (IPN) (*n* = 72).

## Data Availability

The datasets used for this study are available from the corresponding author on reasonable request.

## Abbreviations

AUROC: Area under the receiver operating characteristics curve
BMI: Body mass index
CDF: Catheter-drainage failure
CT: Computed tomography
CTSI: Computed Tomography Severity Index
ICU: Intensive care unit
IPN: Infected pancreatic necrosis
OR: Odds ratio
ROC: Receiver operating characteristics
SOFA: Sequential Organ Failure Assessment
