# Supplementary material for: Infected pancreatic necrosis complicating severe acute pancreatitis in critically ill patients: predicting catheter drainage failure and need for necrosectomy
Source: Ann Intensive Care. 2022 Aug 2;12:71. doi: 10.1186/s13613-022-01039-z (PMC9346045; doi:10.1186/s13613-022-01039-z)
Supplement: Supplementary file 1 — Additional file 1: Digital Content S1. SOFA score (Vincent et al. 1998 [19]). Digital Content S2. Computed tomography (CT) findings according to the revised Atlanta classification [20] and study by Hollemans et al. [8]. Digital Content S3. Dutch nomogram [8]. Digital Content S4. Microorganism recovered from cultures of specimens of infected pancreatic necrosis (IPN); distribution of multidrug-resistant (MDR) microorganisms. Digital Content S5. Antibiotics used before drainage (n = 41) then during the course of infected pancreatic necrosis (IPN) (n = 72). [file 13613_2022_1039_MOESM1_ESM.docx]

**Supplemental Digital Content 1: SOFA score** (Vincent et al., 1998 [19])


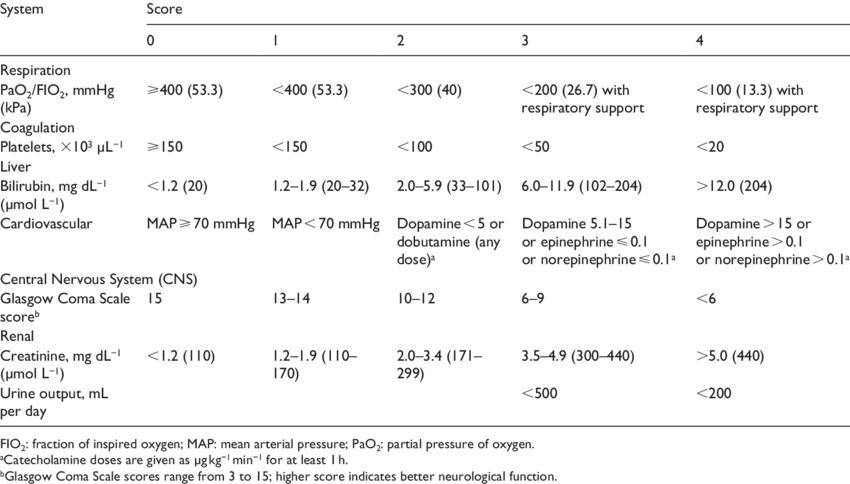


A SOFA score ≥2 in any component defines failure of the relevant organ.

| **Supplemental Digital Content 2: Computed tomography (CT) findings according to the revised Atlanta classification (Banks et al., 2013) and study by Hollemans et al. (2016)** | |
| --- | --- |
| Homogeneous collection | Collection consisting of a single density (i.e., liquid or nonliquid density) |
| Heterogeneous collection | Collection consisting of multiple densities (i.e., liquid and nonliquid densities) |
| Pancreatic necrosis | Focal nonenhancement of the pancreatic parenchyma |
| Extrapancreatic necrosis | Heterogeneous peripancreatic collection(s) persisting after the first week of onset of symptoms, without the presence of pancreatic necrosis |
| Pattern of pancreatic necrosis | |
| Central | Transparenchymal lack of enhancement of pancreatic neck and/or body with a viable pancreatic head and viable upstream tail of at least 3 cm |
| Right sided | Lack of enhancement primarily in the pancreatic head (i.e., at least 50% nonenhancement) without or with minimal necrosis of other parts of the pancreas |
| Left sided | Lack of enhancement primarily in the pancreatic tail with or without involvement of the body and normal enhancement of the head and neck of the pancreas |
| Subtotal | Lack of enhancement of pancreatic neck, body, and greater part of pancreatic head and tail |
| Scattered | Scattered area(s) of lack of enhancement throughout the pancreas, without full thickness or transparenchymal necrosis (i.e., partial-thickness necrosis) |
| Spread pattern of peripancreatic collections | |
| Central | Central area; intraperitoneal in the lesser sac and/or subperitoneal in mesenteries (i.e., small bowel mesenteries and mesocolon transversum) and/or in the retroperitoneum without full extension to the lateroconal fascia |
| Right | Extension to right retrocolic space/retroperitoneum (i.e., right anterior pararenal space, posterior pararenal space, perirenal space—may extend to the extraperitoneal spaces of the pelvis) and at least extending to the lateroconal fascia with or without concomitant involvement of the central area |
| Left | Extension to left retrocolic space or retroperitoneum (i.e. left anterior pararenal space, posterior pararenal space, perirenal space—may extend to the extraperitoneal spaces of the pelvis) and at least extending to the lateroconal fascia with or without concomitant involvement of the central area |
| Bilateral | Extension to both retrocolic spaces or retroperitoneum (anterior pararenal spaces, posterior pararenal spaces, perirenal spaces—may extend to the extraperitoneal spaces of the pelvis) at least extending to lateroconal fascia on both sides with or without concomitant involvement of the central area |
| Degree of  encapsulation | (1) no visible wall  (2) some wall formation;  (3) extensive wall formation;  (4) complete wall (i.e., walled-off necrosis) |
| Definitions of morphological features of acute pancreatitis | |
| APFC (acute peripancreatic fluid collection) | Peripancreatic fluid associated with interstitial edematous pancreatitis with no associated peripancreatic necrosis. This term applies only to areas of peripancreatic fluid seen within the first 4 weeks after onset of interstitial edematous pancreatitis and without the features of a pseudocyst.  Contrast-enhanced CT (CECT) criteria:  ▸ Occurs in the setting of interstitial edematous pancreatitis  ▸ Homogeneous collection with fluid density  ▸ Confined by normal peripancreatic fascial planes  ▸ No definable wall encapsulating the collection  ▸ Adjacent to pancreas (no intrapancreatic extension) |
| Pancreatic pseudocyst | An encapsulated collection of fluid with a well defined inflammatory wall usually outside the pancreas with minimal or no necrosis. This entity usually occurs more than 4 weeks after onset of interstitial edematous pancreatitis.  CECT criteria:  ▸ Well circumscribed, usually round or oval  ▸ Homogeneous fluid density  ▸ No nonliquid component  ▸ Well-defined wall; that is, completely encapsulated  ▸ Maturation usually requires >4 weeks after onset of acute pancreatitis; occurs after interstitial edematous pancreatitis |
| ANC (acute necrotic collection) | A collection containing variable amounts of both fluid and necrosis associated with necrotizing pancreatitis; the necrosis can involve the pancreatic parenchyma and/or the peripancreatic tissues  CECT criteria:  ▸ Occurs only in the setting of acute necrotizing  pancreatitis  ▸ Heterogeneous and nonliquid density of varying degrees in different locations (some appear homogeneous early in their course)  ▸ No definable wall encapsulating the collection  ▸ Location—intrapancreatic and/or extrapancreatic |
| WON (walled-off necrosis) | A mature, encapsulated collection of pancreatic and/or peripancreatic necrosis that has developed a well-defined inflammatory wall. WON usually occurs >4 weeks after onset of necrotizing pancreatitis.  CECT criteria:  ▸ Heterogeneous, with liquid and nonliquid density, with varying degrees of loculations (some may appear homogeneous)  ▸ Well-defined wall, that is, completely encapsulated  ▸ Location—intrapancreatic and/or extrapancreatic  ▸ Maturation usually requires 4 weeks after onset of acute necrotizing pancreatitis |

Banks, Peter A., Thomas L. Bollen, Christos Dervenis, Hein G. Gooszen, Colin D. Johnson, Michael G. Sarr, Gregory G. Tsiotos, Santhi Swaroop Vege, et Acute Pancreatitis Classification Working Group. 2013. Classification of Acute Pancreatitis--2012: Revision of the Atlanta Classification and Definitions by International Consensus. *Gut* 62 (1): 102‑11. <https://doi.org/10.1136/gutjnl-2012-302779>.

Hollemans, Robbert A., Thomas L. Bollen, Sandra van Brunschot, Olaf J. Bakker, Usama Ahmed Ali, Harry van Goor, Marja A. Boermeester, et al. 2016. Predicting success of catheter drainage in infected necrotizing pancreatitis. *Ann Surg.* 263 (4): 787–792.

**Supplemental Digital Content 3: Dutch nomogram (Hollemans et al., 2016)**

Nomogram for the predicting success of catheter drainage to treat infected necrotizing pancreatitis. Each of four predictors is assigned 0, 5, 8, 10, or 12 points. The sum of the numbers lies between 0 and 40 and falls on the “Total points” line. This line synchronizes with the underlying line “Success probability of catheter drainage (%)”, which indicates the chance of successful catheter drainage in infected necrotizing pancreatitis.

Hollemans, Robbert A., Thomas L. Bollen, Sandra van Brunschot, Olaf J. Bakker, Usama Ahmed Ali, Harry van Goor, Marja A. Boermeester, et al. 2016. Predicting success of catheter drainage in infected necrotizing pancreatitis. *Ann Surg.* 263 (4): 787–792.

**Supplemental Digital Content 4:** Microorganism recovered from cultures of specimens of infected pancreatic necrosis (IPN); distribution of multidrug-resistant (MDR) microorganisms

Different bacteria found into IPN

Different multi-drug resistant bacteria found into IPN


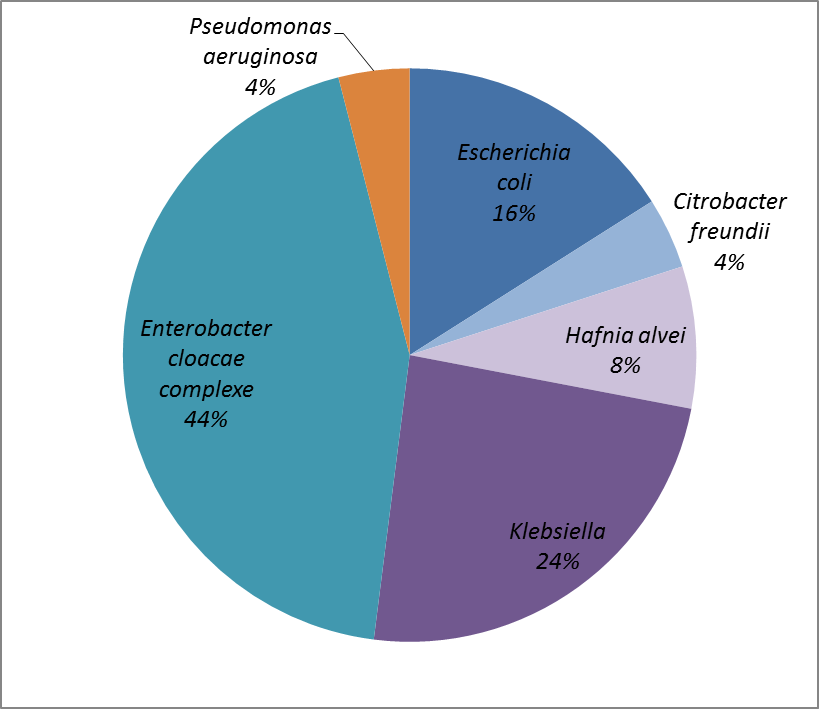

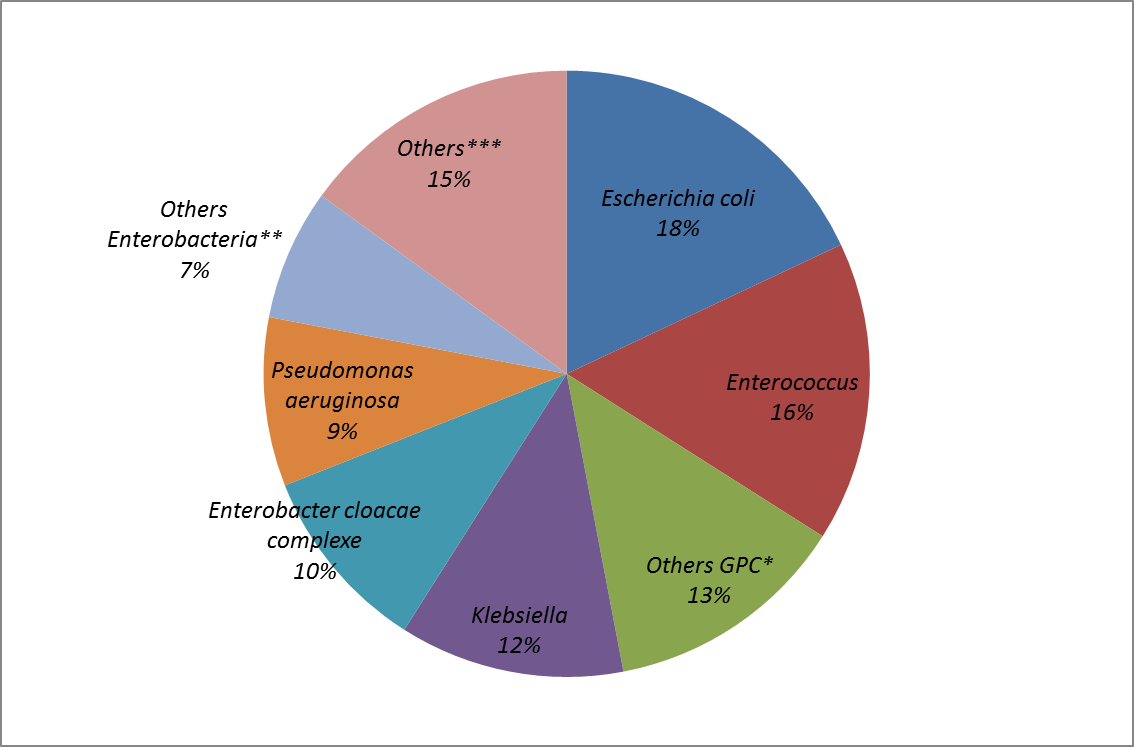


GPC: Gram-positive cocci

*Other GPC: *staphylococci* and *streptococci*

**Other *Enterobacteria*: *Hafnia alvei, Serratia marcescens, Citrobacter freundii,*

***Other: *Stenotrophomonas maltophilia, Lactobacillus, Veillonella, Haemophilus influenzae, Sutterella*


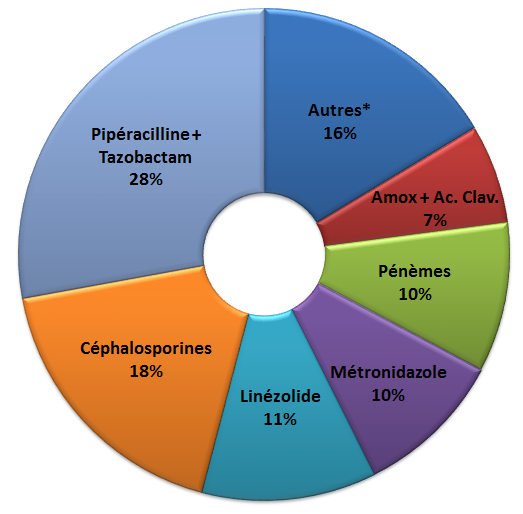

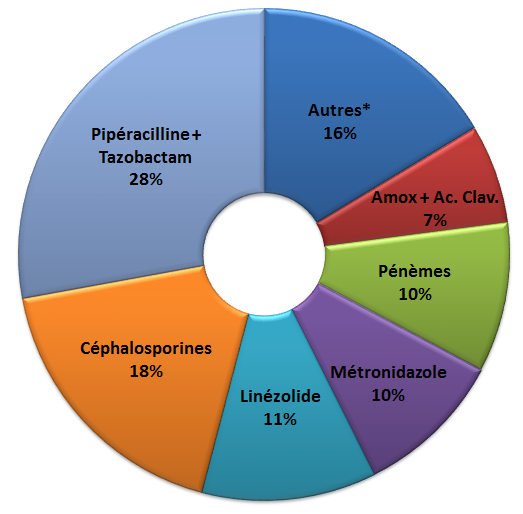

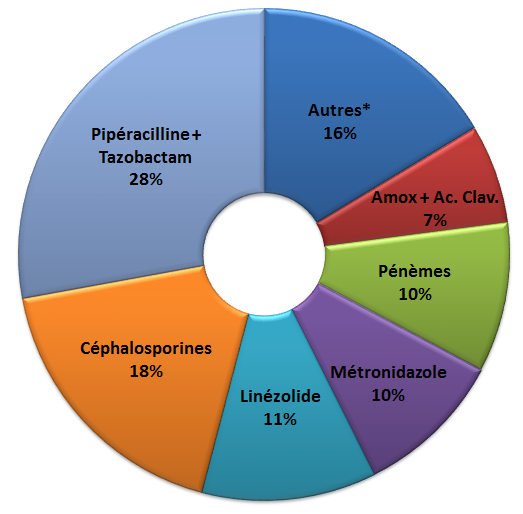


**Supplemental Digital Content 5:** Antibiotics used before drainage (n=41) then during the course of infected pancreatic necrosis (IPN) (n=72)

Different molecules of antibiotics used on the course of IPN (n=70)

Different molecules of antibiotics used prior to drainage (n=41)


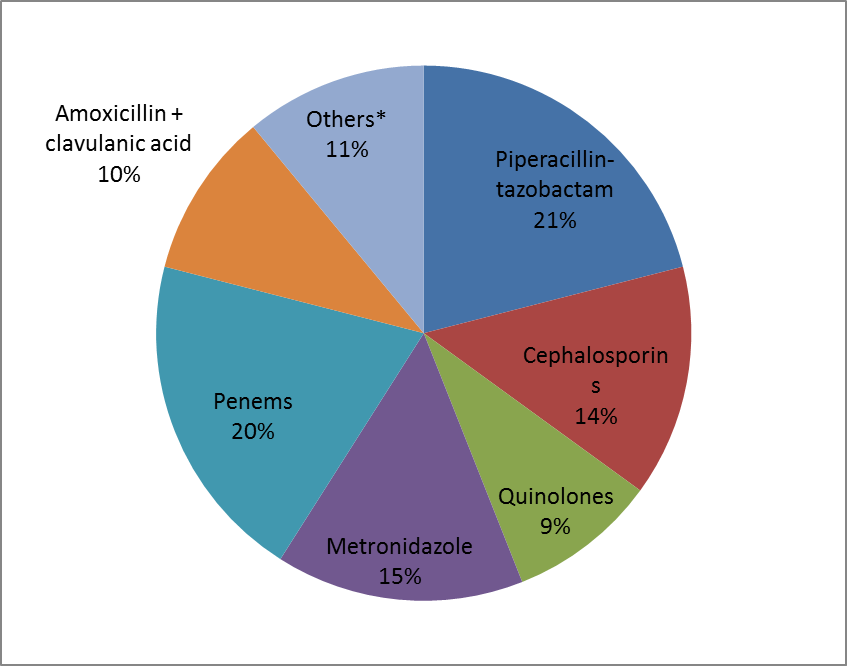

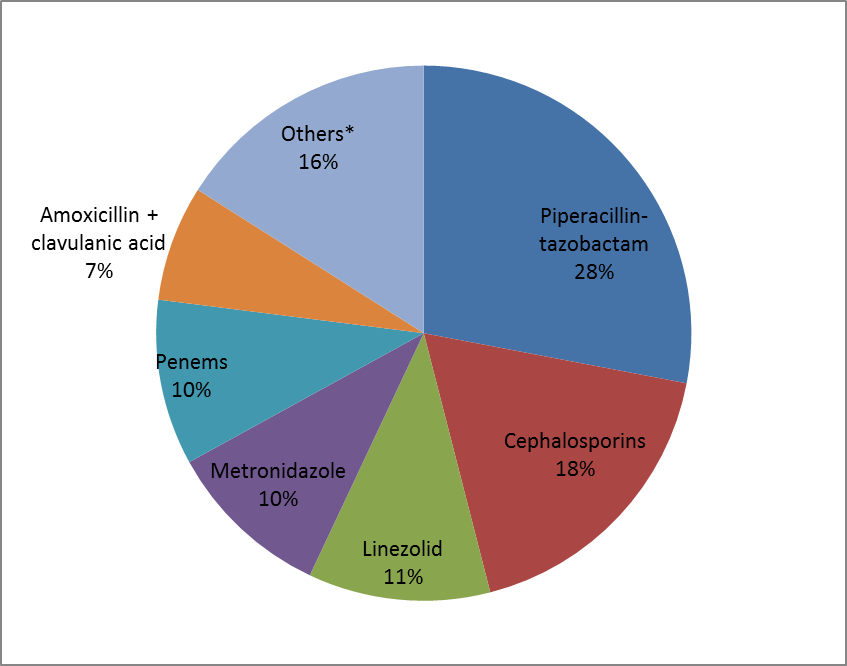


*Other: cloxacillin, colimycin, vancomycin,

sulfamethoxazole, quinolones, amoxicillin, aminoglycosides

*Other: linezolid, sulfamethoxazole, colimycin, vancomycin, oxacillin, amoxicillin, aminoglycosides
